# Influence of Mineralogical Heterogeneity on Acid–Rock Interaction in Dolomitized Carbonates from the Parnaíba Basin, Northeastern Brazil: Implications for Reservoir Matrix Acid Stimulation

**DOI:** 10.1021/acsomega.6c00330

**Published:** 2026-06-03

**Authors:** Jéssica Nascimento Pereira, Natalino da Silva Souza, Igor Alexandre Rocha Barreto, Renato Sol Paiva de Medeiros, José Leão de Luna, Pedro Tupã Pandava Aum, Cláudio Regis dos SantosLucas

**Affiliations:** † Postgraduate Program in Geology and Geochemistry, Geoscience Institute, Federal University of Pará (UFPA), Augusto Corrêa Street s/n, Belém 66075-110, Pará, Brazil; ‡ Laboratory of Integrated Research in Geoenergy, Federal University of Pará (UFPA), Salinópolis 66075-110, Pará, Brazil; § School of Engineering, Federal University of Pará (UFPA), Salinópolis 66075-110, Pará, Brazil; ∥ Petroleum Science and Engineering Laboratory, Federal University of Pará (UFPA), Salinópolis 66075-110, Pará, Brazil

## Abstract

In matrix acid stimulation of carbonate rocks, understanding acid–rock interaction is essential to optimize wormhole formation and overall treatment efficiency, since heterogeneity can lead to distinct dissolution patterns. Mineralogical heterogeneity significantly affects this process due to the different dissolution kinetics of calcite and dolomite; however, it remains unclear how grain size, crystal defects, and mineral distribution influence this interaction. To investigate these factors, five highly dolomitized carbonate samples from the Piauí Formation (Parnaíba Basin) were characterized by petrography, X-ray diffraction, X-ray fluorescence, scanning electron microscopy, and porosity measurements, and subjected to static dissolution in 1 M HCl with evaluation of mass loss and quantification of calcium and magnesium ions. The results showed that texture and mineralogical composition play a fundamental role in dissolution: larger crystals generated wider cavities due to detachment during acid attack; samples with higher Ca and Mg contents relative to Si exhibited higher dissolution rates, whereas increasing quartz content reduced reactivity. Most samples showed an early predominance of Ca^2+^ release followed by increasing Mg^2+^ concentrations, reflecting preferential calcite dissolution and progressive dolomite contribution. Well-developed crystals with fewer structural defects exhibited lower reactivity. Postdissolution peak broadening in XRD patterns suggests structural modification of the minerals. Overall, mineralogical heterogeneity controls acid–rock reactivity across multiple scales, highlighting the importance of integrated mineralogical and textural characterization to improve prediction and optimization of acid stimulation in heterogeneous carbonate reservoirs.

## Graphical abstract

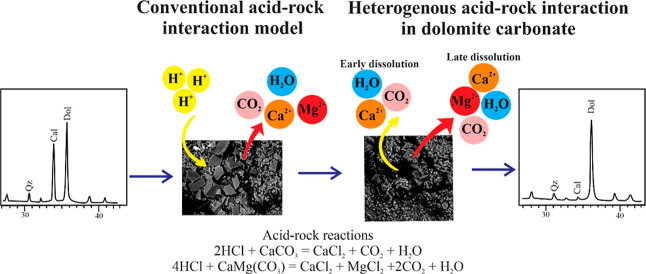

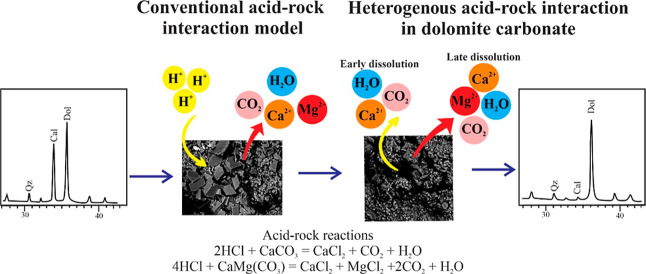

## 1 Introduction

Carbonate reservoirs account for approximately 60% of the world’s known oil reserves and 40% of the known gas reserves, representing a major component of global hydrocarbon production.^1,2^ However, operations such as drilling and cementing may cause formation damage, reducing permeability near the wellbore and decreasing productivity.^3^ In addition, many carbonate reservoirs naturally exhibit low permeability due to intrinsic heterogeneity, which further limits production efficiency.^4^

Matrix acidization is a widely used stimulation technique aimed at removing near-wellbore damage or enhancing permeability in carbonate formations. In this process, acid is injected below fracture pressure, dissolving the rock matrix and creating conductive channels known as wormholes. The efficiency of acid stimulation depends strongly on rock–fluid interactions and on the geological characteristics of the reservoir.^5^

Wormhole formation results from coupled reaction–diffusion–advection processes and rock–fluid interactions, which control acidizing efficiency.^6^ Hydrochloric acid (HCl), typically used about 15 wt %, is the most common acid for carbonate stimulation. However, at high temperatures, organic acids such as acetic and formic acids are often preferred due to corrosion concerns.^7^

Carbonate rocks are inherently heterogeneous. Variations in mineral composition (e.g., calcite and dolomite), grain size, crystal texture, and spatial organization influence reactive surface area and dissolution pathways. Calcite and dolomite exhibit distinct kinetic behaviors during acid reaction, with calcite dissolving more readily in HCl at low temperatures, whereas dolomite requires higher temperatures to achieve comparable dissolution rates.^8^ Although several studies have investigated dissolution kinetics of individual carbonate minerals,^9–13^ fewer studies have addressed how intrinsic mineralogical and textural heterogeneity at the rock scale controls dissolution behavior under acid stimulation conditions. Understanding these controls is essential for improving acidizing efficiency and minimizing treatment failure, particularly in heterogeneous carbonate systems.

Therefore, the main objective of this study is to evaluate how mineralogical and textural heterogeneity controls the reactivity of carbonate rocks under acid stimulation conditions. To achieve this, petrographic analyses, mineralogical characterization, and controlled laboratory dissolution experiments were performed in order to correlate rock fabric features with dissolution rates and reactive behavior. The results aim to provide a better understanding of mineralogical framework influence on acid–rock interaction and to contribute to the optimization of stimulation strategies in heterogeneous carbonate reservoirs.

Carbonate samples from the Mocambo carbonates of the Piauí Formation (Parnaíba Basin, Brazil) were selected due to their significant facies and diagenetic heterogeneity, including dolomitization, recrystallization, and dissolution features that directly affect rock–fluid interaction.

## 2 Geological Setting

The Parnaíba Basin is an intracratonic basin located in the northwestern portion of northeastern Brazil.^14^ It covers an area of approximately 600,000 km^2^, with a depocenter containing rocks up to 3500 m thick.^15^ The basin lies in the western part of northeastern South America, has an ellipsoidal shape, and is bounded to the north by the São Luís–Grajaú and Barreirinhas basins, to the northwest by the Marajó Trough, and to the south and southeast by extensive Precambrian basement outcrops.^16^ Its formation occurred during the Stabilization Stage of the South American Platform, when large syneclises developed, resulting in the formation of intracratonic sedimentary basins in remote interior regions, separated by uplifted areas of the Precambrian basement.^17^

According to Vaz et al.,^18^ the Parnaíba Basin is subdivided into five depositional supersequences bounded by unconformities: the Silurian Sequence, Serra Grande Group; the Middle Devonian–Early Carboniferous Sequence, Canindé Group; the Late Carboniferous–Early Triassic Sequence, Balsas Group; the Jurassic Sequence, Pastos Bons Formation; and the Cretaceous Sequence, including the Codó, Corda, Grajaú, and Itapecuru Formations.

The Balsas Group, which comprises the Piauí, Pedra de Fogo, Motuca, and Sambaíba formations, was deposited during a period of significant environmental and tectonic changes in the basin. During this time, open-marine settings with wide circulation and temperate climate gave way to restricted, shallow, hot, and arid environments due to a regression from the Permian to the Early Triassic, which led to progressive desertification of the basin.^18^

The Piauí Formation reaches a maximum thickness of approximately 330 m.^19^ It occurs in the southern and northeastern portions of the Parnaíba Basin, overlying the Poti Formation and being overlain by the Pedra de Fogo Formation.^20,21^ Mesner and Wooldridge^22^ divided this formation into a Lower Member, composed of pink to red sandstones and red shales, and an Upper Member, composed of red and green shales, anhydrite, and fossiliferous carbonates.

The Piauí Formation has been interpreted as representing shallow-marine, aeolian (arid conditions), and fluviodeltaic deposits.^21,23,24^ According to Anelli^25^ this formation contains a diverse marine invertebrate fauna, including bivalves, gastropods, cephalopods, brachiopods, trilobites, and bryozoans, which are associated with a carbonate platform environment.

Campanha and Rocha Campos^26^ identified, in the Mocambo carbonate sequence of the Upper Member of the Piauí Formation, the presence of microgastropods, microbivalves, and fragments of corals and algae. Dias et al.^27^ reported three conodont species from carbonates of the Mocambo sequence (Diplognathodusorphanus, Idiognathodus incurvus e Adetognathus lautus) suggesting a Pennsylvanian age and a very shallow marine platform environment, with facies ranging from infratidal to intertidal. This sequence crops out at Fazenda Mocambo and at the Icaraí Mining Company, both located in the municipality of José de Freitas, Piauí State, and corresponds to the sampling points Mo-01 and Mo-04 analyzed in this study, as shown in Figure 1.

**Figure 1:**
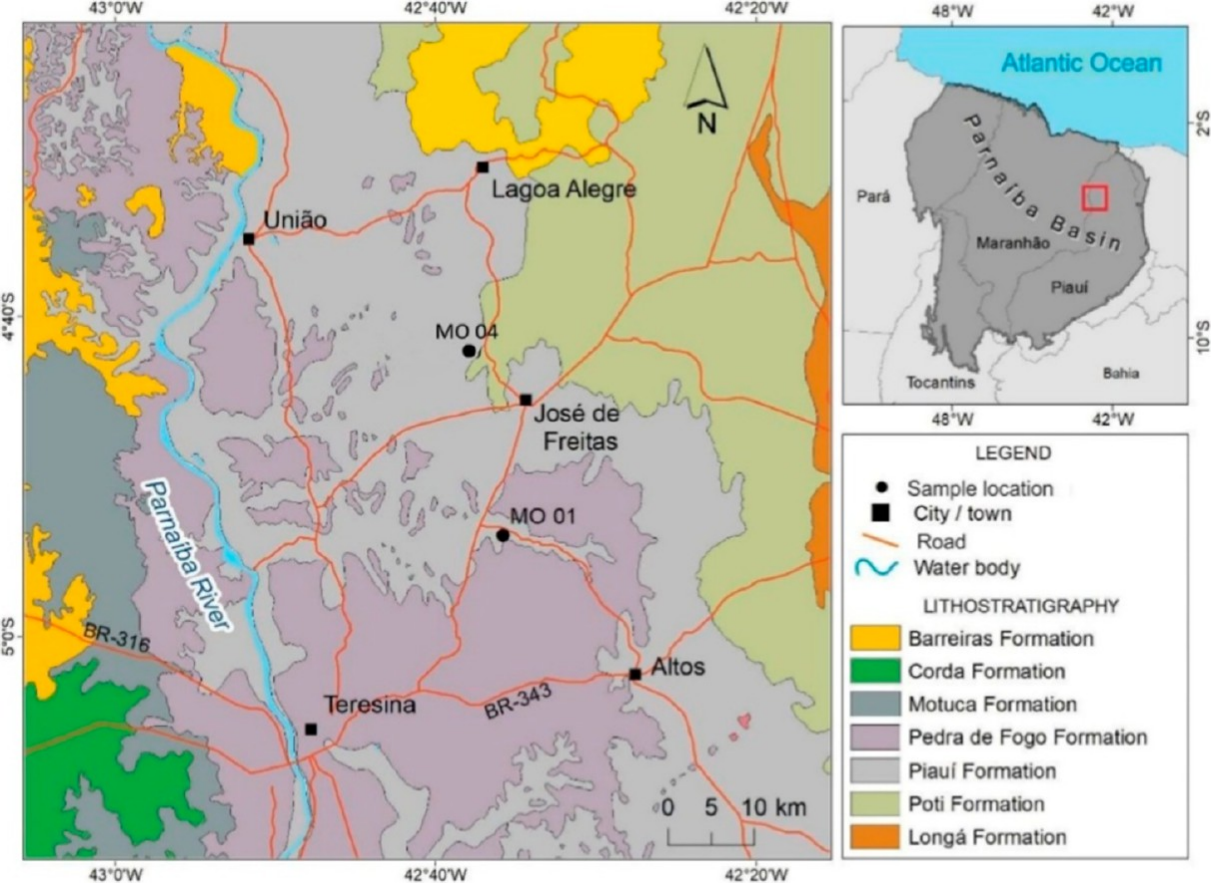
Location map of the sample collection points. Source: adapted with permission from Dias at al., Journal of South American Earth Sciences, 2022. Copyright 2022 Elsevier Ltd.

## 3 Materials and Methods

In this study, five carbonate rock samples were collected from outcrops located in the northwestern portion of the State of Piauí, in the municipality of José de Freitas, corresponding to the upper section of the Piauí Formation. Part of the samples was reserved for chemical and mineralogical analyses and for the preparation of petrographic thin sections, while the remaining material was used to prepare 30 and two mini-plugs (eight for each sample), approximately 1.0 cm in height and 0.8 cm in diameter. The methodology applied to the mini-plugs is summarized in the diagram shown in Figure 2.

**Figure 2:**
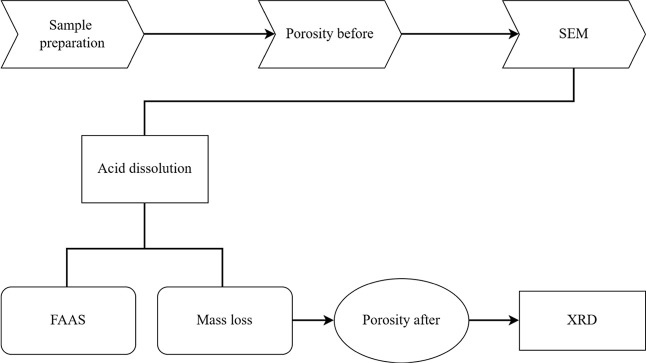
Experimental setup applied to the mini-plugs.

### 3.1 Physicochemical Analyses

For the petrographic study, six thin sections were prepared at the Lamination Workshop of the Federal University of Pará (Belém, Pará, Brazil). The carbonate rock sections were stained with Alizarin Red S (0.2 g/100 mL of 1.5% HCl) to distinguish calcite from dolomite at the Sedimentology Laboratory of the Federal University of Pará (Belém, Pará, Brazil), following the method proposed by Dickson.^28^ Based on the stained thin sections, petrographic descriptions were performed considering the main constituents of the rocks, such as matrix, cement, bioclasts, siliciclastic grains, and pores. The classification of the carbonate rocks followed the criteria established by Wright.^29^ Grain size measurements correspond to the longest measurable axis of individual grains observed in thin section. This criterion was applied consistently to all reported grain-size estimations.

The chemical and mineral compositions were determined by X-ray fluorescence (XRF) and X-ray diffraction (XRD) analyses at the Mineral Characterization Laboratory (LCM) of the Federal University of Pará (Belém, Pará, Brazil), using the powder method. For XRD analysis, an Empyrean PANalytical diffractometer equipped with a Co anode (Kα1 = 1.789010 Å) was used, and the results were processed using the X’Pert HighScore Plus software (version 3.0.0), which enables comparison with data from the International Center for Diffraction Data (ICDD) database. The XRF analyses were performed with a PANalytical Axios Minerals spectrometer equipped with a rhodium (Rh) anode, and data acquisition and processing were carried out using the SuperQ Manager software, version 5.3 (PANalytical).

High-resolution images of one face of each mini-plug were obtained before and after the reactivity experiments by scanning electron microscopy (SEM) using the backscattered electron technique, in order to observe mineral zoning and surface textures. Additionally, semiqualitative point chemical analyses were performed by energy dispersive X-ray spectroscopy (EDS), coupled to the SEM equipment. The analyses were conducted at the Microanalysis Laboratory of the Federal University of Pará (Belém, Pará, Brazil), using a Zeiss scanning electron microscope, model Leo 1430.

The porosities (ϕ) of the mini-plugs were measured before and after the acid experiments using the saturation technique recommended by ISRM,^30^ following eq 1, which expresses the relationship between pore volume and total volume (*V*_t_). The mini-plugs were saturated with ultrapure water (UPW; conductivity = 0.05 μS/cm, resistivity = 18.18 MΩ cm) in a DCI MSC-2000 saturator, remaining under a vacuum pressure of 25 in Hg for 2 h and under pressure saturation (2000 psi) for 20 h. The pore volume was determined from the difference between the masses of the saturated (*m*_sat_) and dry (*m*_s_) samples divided by the specific mass of water (ρ_w_), which was determined by pycnometry.

1 $$\varphi =\frac{\left(m_{sat}-m_{s}\right)}{\rho_{w}V_{t}}\times 100\%$$

### 3.2 Reactivity Analysis

The mini-plugs were dried in an oven at 150 °C for 1 h and cooled in a desiccator containing silica gel for an additional hour, then weighed on an analytical balance to determine their initial mass.

The *T*_50_, parameter, which represents the dissolution time required to reduce the sample mass by 50%, was determined for each of the five samples according to Jora et al.^9^ by linear estimation after a sufficiently long reaction period. Mass-loss analyses were performed at time intervals corresponding to multiples of 1/4 *T*_50_, i.e., (1/4, 1/3, 1/2, and *T*_50_) using one mini-plug for each dissolution period (see Table 1) following a modified methodology based on Jora et al.^9^ Duplicate experiments were carried out for each dissolution time (see Supporting information Figure S1). The resulting solutions from the reactions were collected for calcium and magnesium cation analyses. In each test, 40 mL of 1 mol/L HCl solution was used. At the end of the predetermined reaction time, the rocks were rinsed with ultrapure water (UPW), oven-dried at 150 °C for 5 h, cooled to room temperature for 1 h in a desiccator, and subsequently weighed on an analytical balance.

**Table 1:** Dissolution Time (min)

samples	1/4	1/3	1/2	*T*_50_
Mo-01	30.73	61.47	92.20	121.76
Mo-01-B	27.57	55.13	82.70	109.36
Mo-04-B	33.73	67.47	101.20	136.76
Mo-04-C	25.32	50.63	75.95	108.76

Calcium and magnesium concentrations in the solutions were determined by flame atomic absorption spectroscopy (FAAS), using an analytical calibration curve (blank plus five standard concentrations) prepared from inorganic standard solutions. A 1 M KCl solution was used as an ionization suppressor. The acidity of the samples and standard solutions was adjusted to 0.5% using HCl. Detection (LD) and quantification (LQ) limits were calculated as 3σ/α and 10σ/α, respectively, where σ is the standard deviation of ten blank measurements and α is the slope of the calibration curve. The LD values ranged from 1.95 × 10^–6^ mol/L for Ca and 4.94 × 10^–7^ mol/L for Mg, while the LQ values ranged from 6.51 × 10^–6^ mol/L for Ca and 1.60 × 10^–6^ mol/L for Mg. The accuracy of the method was assessed through spike-and-recovery tests using three standard concentrations (6, 10, and 14 mg/L) added to randomly selected samples. Recoveries were 134%, 96%, and 110% for Ca and 97%, 80%, and 70% for Mg.

After the dissolution tests, XRD analyses were repeated on two mini-plugs from each sample, corresponding to the 1/4 and *T*_50_ reaction times.

## 4 Results and Discussion

### 4.1 Sample Characterization

#### 4.1.1 Petrographic Analysis

The analyzed samples were collected from outcrops of the Piauí Formation, Parnaíba Basin, located in the municipality of José de Freitas, Piauí State. The outcrop occurs in both active and inactive quarry fronts as well as in the urban area, with a total thickness of up to 55 m. The exposures exhibit carbonate and siliciclastic facies and microfacies that compose the upper portion of the formation.^21^

The samples used in this study were collected from two localities: Fazenda Contenda, within the mining area of Mineradora Icaraí (samples Mo-04, Mo-04-B, and Mo-02), and Fazenda Mocambo (samples Mo-01 and Mo-01-B). The latter is noteworthy for having given rise to the informal denomination of the studied carbonates, as described by.^31^ The samples correspond to a facies association of shallow-marine deposits.^21,27^ In the Mocambo outcrop, these carbonates exhibit a thickness of approximately 1 m, whereas at Fazenda Contenda they reach up to 4 m.

#### 4.1.2 Cementstone

The analyzed thin sections are classified as cementstone according to the classification proposed by Wright,^29^ as they correspond to diagenetic rocks predominantly composed of cement. Terrigenous and allochemical particles were identified as secondary components. The microfacies association comprises dolomite with peloids and quartz (Dpq), dolomite with cnidarians (Dc), and dolomite with quartz (Dq). In general, the thin sections of this association display intense dolomitization, which hindered the detailed identification of bioclasts.

#### 4.1.3 Dolomite with Peloids and Quartz (Dpq)

Samples Mo-01 and Mo-01-B belong to the dolomite with peloids and quartz (Dpq) microfacies. The microfacies shows a matrix predominantly composed of microcrystalline and xenotopic dolomite (∼10 μm). Localized precipitation of iron-oxide cement is also observed at specific points in the thin sections (Figure 3A)

**Figure 3:**
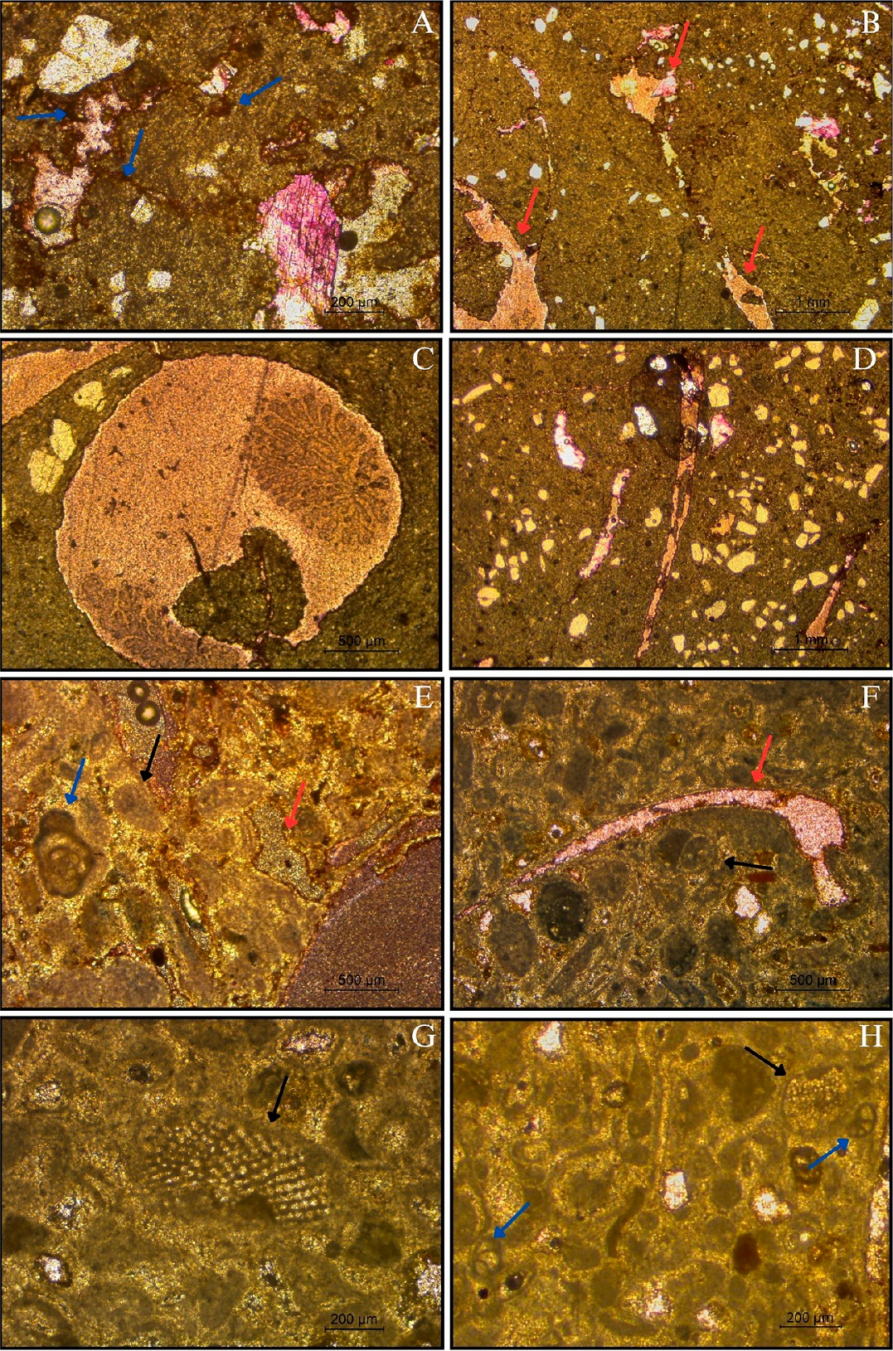
Photomicrographs of the Dolomite with Peloids and Quartz (Dpq) microfacies: (A–D) correspond to sample Mo-01, where (A) cement calcite occurs in different stages of crystallization, microcrystalline and sparry (darker pink), with localized iron-oxide precipitation indicated by arrows; (B) microcrystalline dolomitic matrix showing dispersed quartz crystals and vug pores filled with microcrystalline calcite cement, as indicated by arrows; (C) bioclast filled with microcrystalline calcite cement; (D) elongated undifferentiated bioclastic fragments filled with microcrystalline and sparry calcite cement. (E–H) correspond to sample Mo-01-B, where (E) cross-polarized light, foraminifer (blue arrow), peloid with dolomitic fringe (black arrow), and vug pore filled with microcrystalline dolomite (red arrow); (F) plane-polarized light, bivalve filled with microcrystalline calcite cement at the center, gastropod (black arrow); (G) green algal fragment in the center; (H) plane-polarized light, showing a microcrystalline dolomitic matrix with bioclasts such as foraminifers (blue arrows) and na echinoderm (red arrow), and numerous peloids.

The carbonate framework is composed of quartz grains and bioclastic fragments. In sample Mo-01, the quartz grains are monocrystalline (defined here as single-crystal and no evidence of internal subgrain boundaries), distributed throughout the thin section, and exhibit abrupt extinction under cross-polarized light. Their morphology varies from subangular to subrounded, with grain sizes between 125 and 250 μm, corresponding to fine to medium-grained sand (Figure 3B). Some quartz grains show evidence of dissolution along their margins. Bioclasts are less abundant than quartz grains and include undifferentiated bioclastic fragments ranging from 1000 to 4000 μm in size, which are both micritized and filled with sparry calcite cement (Figure 3C e D).

In sample Mo-01-B, the framework is predominantly composed of peloids approximately 200 μm in diameter, surrounded by fringes of dolomitic cement. Bioclastic grains are frequent and include fragments of bivalves, echinoderms, foraminifers, gastropods, ostracods, and green algae (Figure 3E, F, G e H). These bioclasts range in size from 200 to 4500 μm and are mostly micritized and dolomitized.

Regarding porosity, both primary and secondary porosity were identified. The primary porosity consists of intercrystalline pores observed in areas with microcrystalline dolomite cementation. The secondary porosity includes intragranular and vug pores. Vug pores, common in the samples, are partially filled with sparry calcite (100–500 μm) cement and may be interconnected, forming cavernous pores filled with microcrystalline calcite (∼10 μm); in some cases, these pores are partially filled with iron-oxide cement. Intragranular pores occur within bioclasts.

#### 4.1.4 Dolomite with Cnidarians (Dc)

The Dc microfacies is represented by samples Mo-04-B and Mo-04-C and can be petrographically described as being predominantly composed of xenotopic to hypidiotopic microcrystalline dolomite (∼10 μm), exhibiting low visible porosity. Localized iron-oxide cementation is also observed in some areas (Figure 4A)

**Figure 4:**
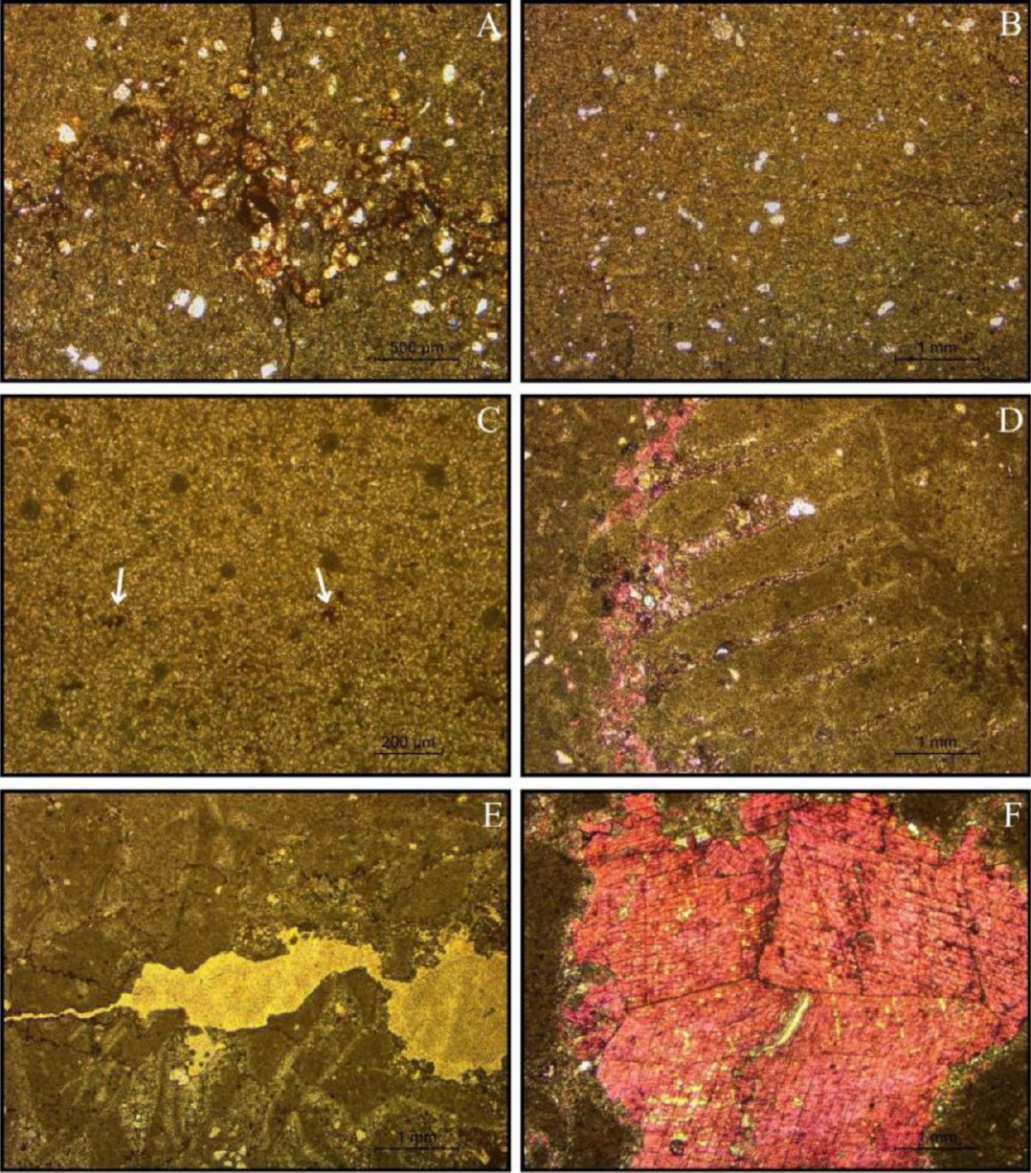
Photomicrographs of the Dolomite with Cnidarians (Dc) microfacies under parallel nicols: (A) quartz grain within a dolomitic matrix, with reddish-brown iron oxide observed at the center; (B) dispersed quartz grains in a dolomitic matrix; (C) dolomitic matrix with peloids showing localized iron-oxide precipitation (white arrows); (D) fragment of a rugose cnidarian; (E) vug pore filled with micrite at the center; (F) sparry calcite crystals.

The carbonate framework consists of quartz grains, bioclasts, and peloids (Figure 4B e C). The quartz grains are monocrystalline, with morphology ranging from subangular to subrounded and grain sizes between 65 and 200 μm, corresponding to very fine- to fine-grained sand. These grains are dispersed throughout the sections and do not exhibit contact with one another.

Bioclasts are scarce, mostly micritized and undifferentiated, although fragments of rugose cnidarians measuring up to 9000 μm filled with sparry calcite cement were identified in sample Mo-04-C (Figure 4D). The peloids have an average grain size of approximately 100 μm and are homogeneously distributed throughout the thin sections.

The observed porosity is low, comprising both primary and secondary porosity. The primary porosity includes a few intercrystalline pores, whereas the secondary porosity corresponds to vug pores. Additionally, dissolution channels extending into areas with concentrations of microcrystalline calcite (∼10 μm) are observed (Figure 4-E). Dissolution seams are also present, appearing as irregular lines with an electrocardiogram-like pattern, as well as regions filled with sparry calcite (Figure 4-F), with crystal sizes ranging from 100 to 3500 μm.

Tables 2, 3, and 4 provide a summary of the petrographic information on the analyzed samples. Table 2 compiles data on the fossils identified in the samples; Table 3 presents the grain sizes of the main constituents; and Table 4 describes the presence of depositional mud and iron oxide.

**Table 2:** Summary of Fossil Occurrences in the Analyzed Samples

Sample	microfacies	presence of fossils	main fossils identified
Mo-01	Dpq	common	undifferentiated bioclastic
Mo-01-B	Dpq	abundant	bivalves; echinoderms, foraminifers, gastropods, ostracods, green algae and undifferentiated bioclastic;
Mo-04-B	Dc	rare	undifferentiated bioclastic;
Mo-04-C	Dc	rare	rugose cnidarians and undifferentiated bioclastic

**Table 3:** Grain Size of the Main Constituents of the Analyzed Samples

sample	calcite: bioclasts (grain size)	calcite: spastic cement (grain size)	dolomite: matrix (grain size)	dolomite: bioclasts (grain size)	quartz (grain size)
Mo-01	1000–4000 μm	100–500 μm	∼10 μm	-	125–250 μm
Mo-01-B	200–4500 μm	100–200 μm	∼10 μm	200–500 μm	-
Mo-04-B	-	-	∼10 μm	∼1000 μm	65–200 μm
Mo-04-C	1000–9000 μm	1000–3500 μm	∼10 μm	∼1000 μm	65–200 μm

**Table 4:** Occurrence of Depositional Mud and Iron Oxide in the Analyzed Samples

sample	depositional carbonate mud	iron oxide
Mo-01	not observed	localized at specific points
Mo-01-B	not observed	around some grains
Mo-04-B	not observed	localized in some areas
Mo-04-C	not observed	localized in some areas

#### 4.1.5 Chemical and Mineralogical Characterization

In the XRD diffractograms obtained (Figure 5), all samples show similar mineralogical compositions, with characteristic peaks of dolomite (Dol) and quartz (Qz). Calcite (Cal) was detected in four samples but was not observed in sample Mo-04-B. Dolomite is the predominant mineral, displaying peaks of higher relative in tensity and high Frequency throughout the entire analyzed 2θ range, which may indicate a greater abundance compared to the other minerals. A visual comparison among the diffractograms shows that, despite small variations in relative intensities, the peak pattern is consistent across all samples, confirming their mineralogical similarity.

**Figure 5:**
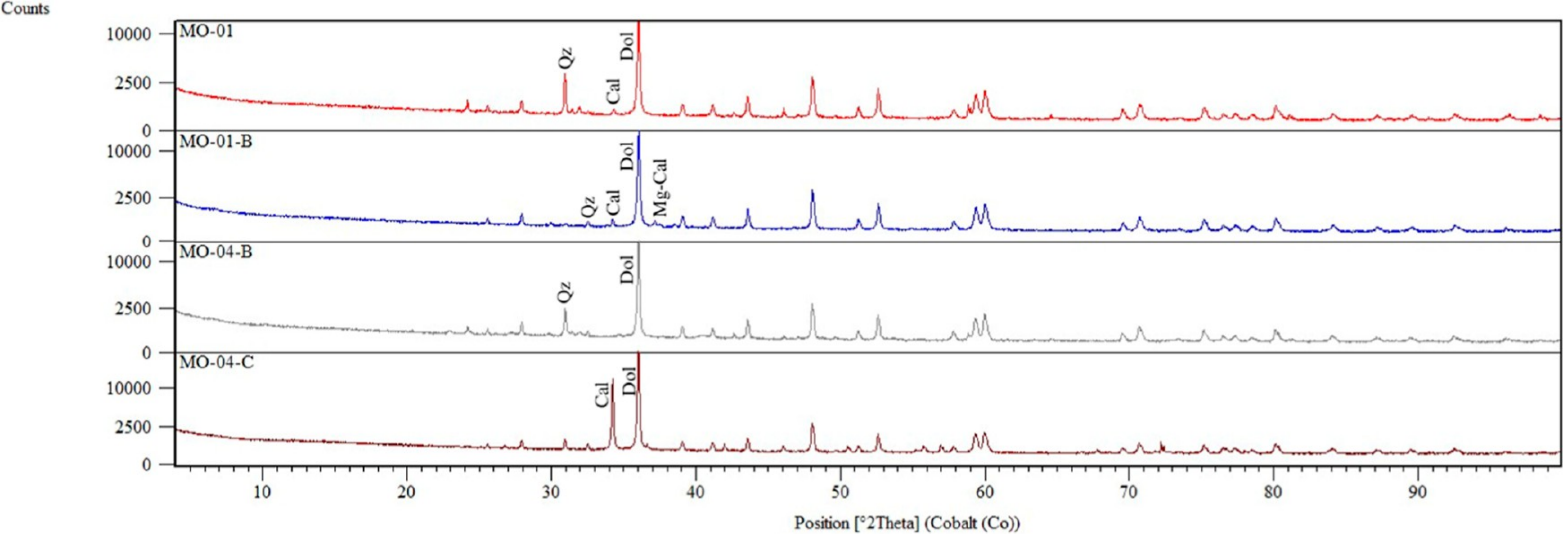
Mineralogical comparison of the samples obtained by X-ray diffraction (XRD).

Figure 6 shows the chemical composition of the five samples based on XRF analyses. A high loss on ignition (LOI) is observed, ranging from 43.6% to 37.3% among the samples, due to the release of CO_2_ from the carbonate minerals. Calcium (Ca) and magnesium (Mg) are dominant in all samples, ranging from 32.9% to 22.4% and from 20.1% to 15.7%, respectively. These high contents are explained by the abundance of dolomite and, to a lesser extent, calcite in the samples. The third most abundant element is silica (Si), ranging from 15.3% to 2.5%, probably derived from quartz, which was identified in all samples through XRD analysis.

**Figure 6:**
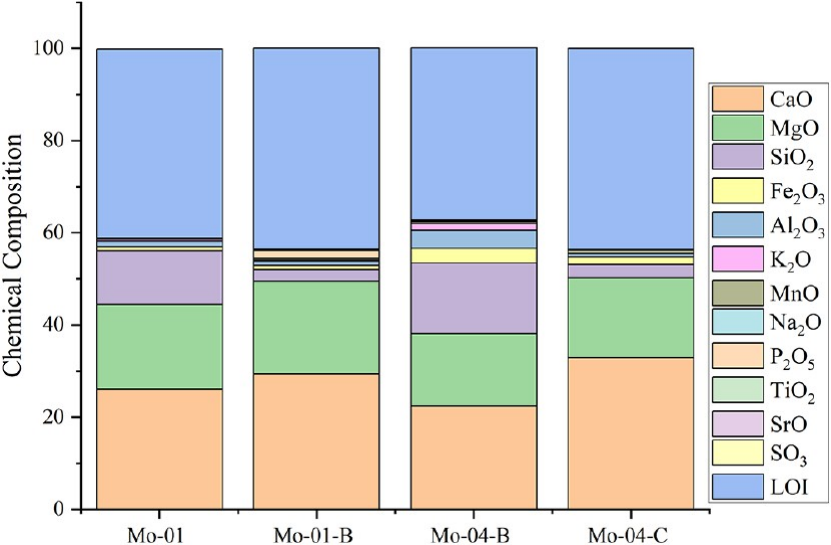
Elemental composition as oxide form obtained by X-ray fluorescence (XRF).

Figure 7 shows the relationship between CaO and MgO contents and SiO_2_ in the samples, comparing the quartz contents with those of dolomite and calcite. It can be observed that samples Mo-01 and Mo-04-B have the highest SiO_2_, contents, which likely indicates a greater amount of quartz. However, while the SiO_2_ content in sample Mo-04-B is balanced with the CaO and MgO contents derived from carbonate minerals, in sample Mo-01 the CaO and MgO levels are higher. The other samples show low SiO_2_ contents and high CaO and MgO contents, implying a lower quartz content relative to dolomite and calcite in these samples. According to Rodríguez et al.^32^ siliceous minerals can influence how the rock surface is affected by dissolution.

**Figure 7:**
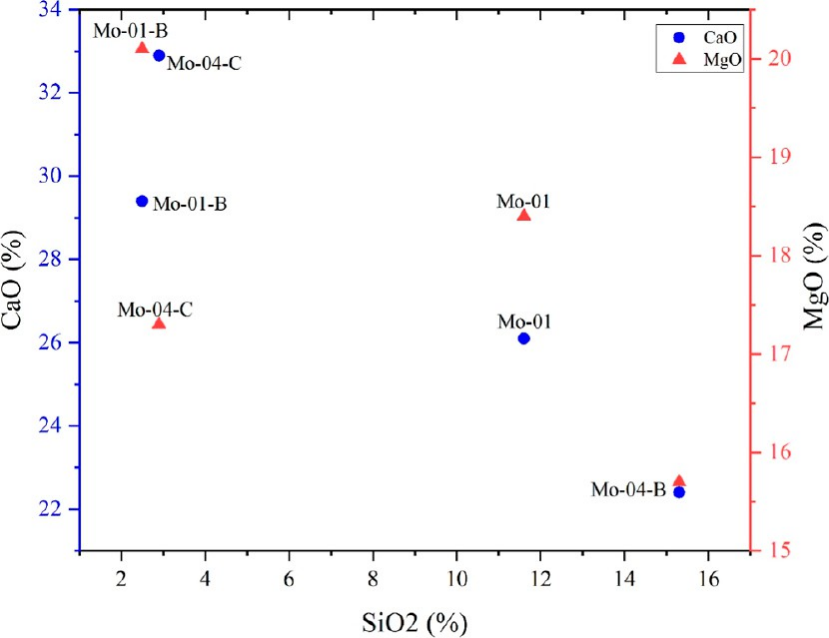
Relation of sample compositions: CaO and MgO vs SiO2

#### 4.1.6 Micromorphological Analyses

SEM images of one face of each mini-plug enabled a qualitative analysis of the morphological characteristics of the samples (Figure 8): Mo-01 (A, B), Mo-01-B (C, D), Mo-04-B (E, F), and Mo-04-C (G, H). In the nonporous regions (A, C, E, G), a homogeneous composition is observed, consisting of anhedral crystals with poorly defined boundaries and a very fine texture, which appears more irregular in A and I.

**Figure 8:**
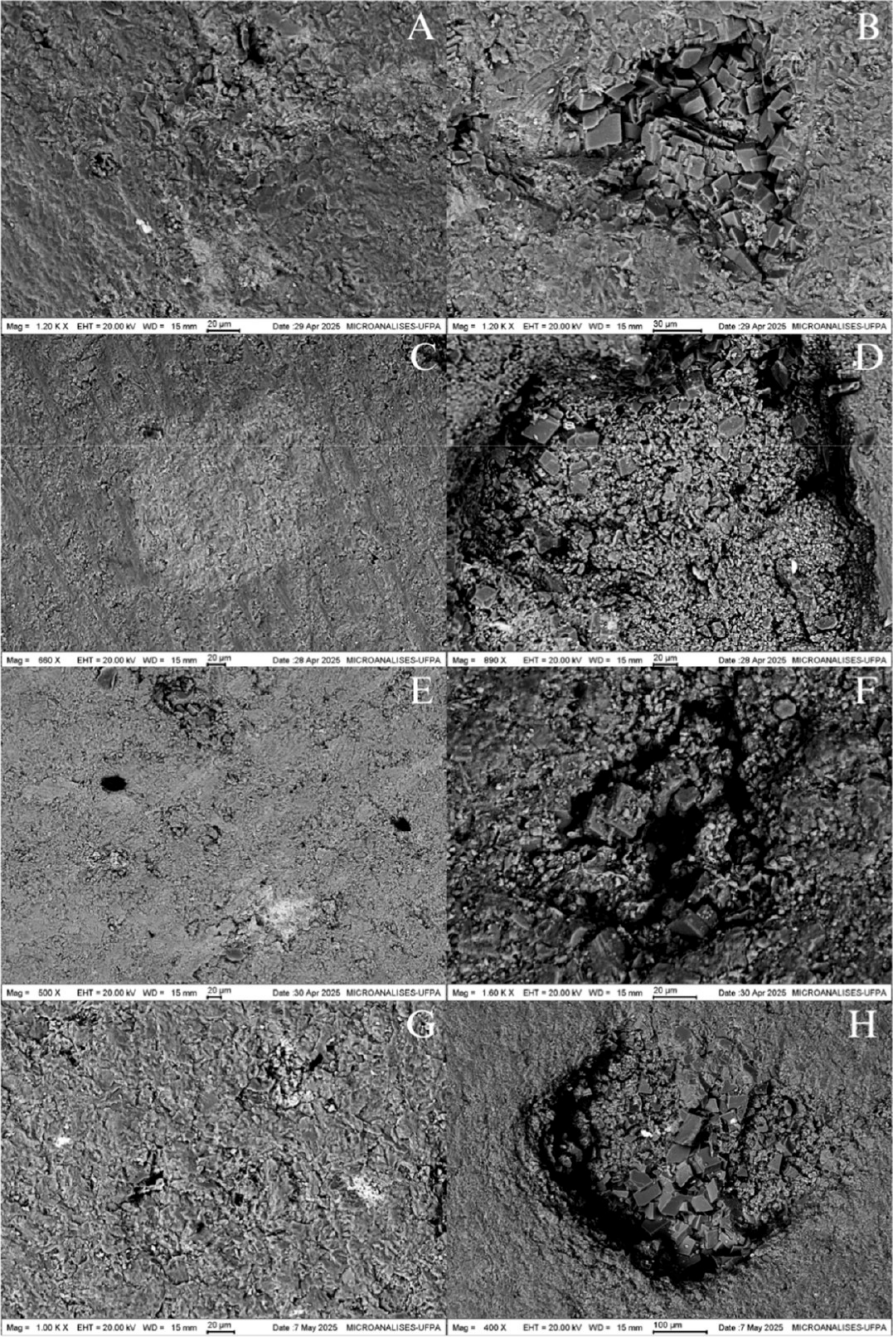
SEM images: A and B Mo-01; C and D Mo-01-B; G and H Mo-04-B; I and J Mo-04-C.

The rhombohedral crystal habit is commonly observed in dolomites.^33^ This habit was identified in the porous regions (B, D, F, H) of the analyzed samples. Sample Mo-01 (B) exhibits well-developed crystals with tabular and rhombohedral habits. In samples Mo-01-B (D), the matrix is predominantly composed of very fine, anhedral to subhedral crystals that are poorly individualized, with only a few crystals showing well-defined boundaries. In samples Mo-04-B (F) and Mo-04-C (H), very fine and irregular anhedral crystals predominate, accompanied by larger crystals that are subhedral in F and range from subhedral to euhedral in H. Although the crystals in images F and H appear to be of similar size, those in J are actually larger, as the magnification used was significantly lower.

It is essential to consider the effects of rock microstructures in acidification and stimulation studies, since experimental results within the same study may vary significantly due to microstructural differences between samples. Moreover, natural rocks are influenced by several complex factors capable of producing marked changes in their microstructure, both between different strata and within distinct regions of the same stratum.^34^

### 4.2 Acid Dissolution

Progressive reduction in sample diameter between 1/4 and *T*_50_ was observed. Samples Mo-01 and Mo-01-B (Dolomite with Peloids and Quartz, Dpq) and Mo-04-B (Dolomite with Cnidarians, Dc) exhibited relatively homogeneous surface dissolution, although Mo-01 and Mo-01-B developed greater surface roughness. In contrast, Mo-04-C (Dc) formed larger cavities. Samples with initially larger crystals tended to develop deeper cavities, likely due to crystal detachment during dissolution (see Supporting Information Figure S2).

According to Li et al.,^35^ the main factors controlling the nonuniform surface morphology of acid corrosion are rock heterogeneity and the nonhomogeneous distribution of the acid fluid. The mineralogical distribution influences the corrosion patterns on the surface, which are also affected by surface roughness.^36^ In the experiments conducted in this study, the distribution of acid around the rock surface was heterogeneous only in the region where the sample was in contact with the bottom of the glass vessel. Therefore, the resulting surface morphology of corrosion is predominantly controlled by the intrinsic heterogeneity of the rock, including mineralogical variations, heterogeneous distribution of minerals on the surface, and the initial roughness of the sample.

Figure 9 shows the porosity of the mini-plugs before and after dissolution. An increase in porosity ranging from 0.31% to 5.87% is observed in the samples of Dolomite with Peloids and Quartz (Dpq) microfacies, although some samples, such as Mo-04-B and Mo-04-C, both assigned to the Dolomite with Cnidarians (Dc) microfacies, show an apparent reduction. In Mo-04-C, the larger cavities formed on the surface do not retain water during the saturation-based measurement, explaining the recorded decrease in porosity. In Mo-04-B, the homogeneous dissolution suggests that the largest pores were concentrated in the dissolved region, also accounting for the reduction in porosity.

**Figure 9:**
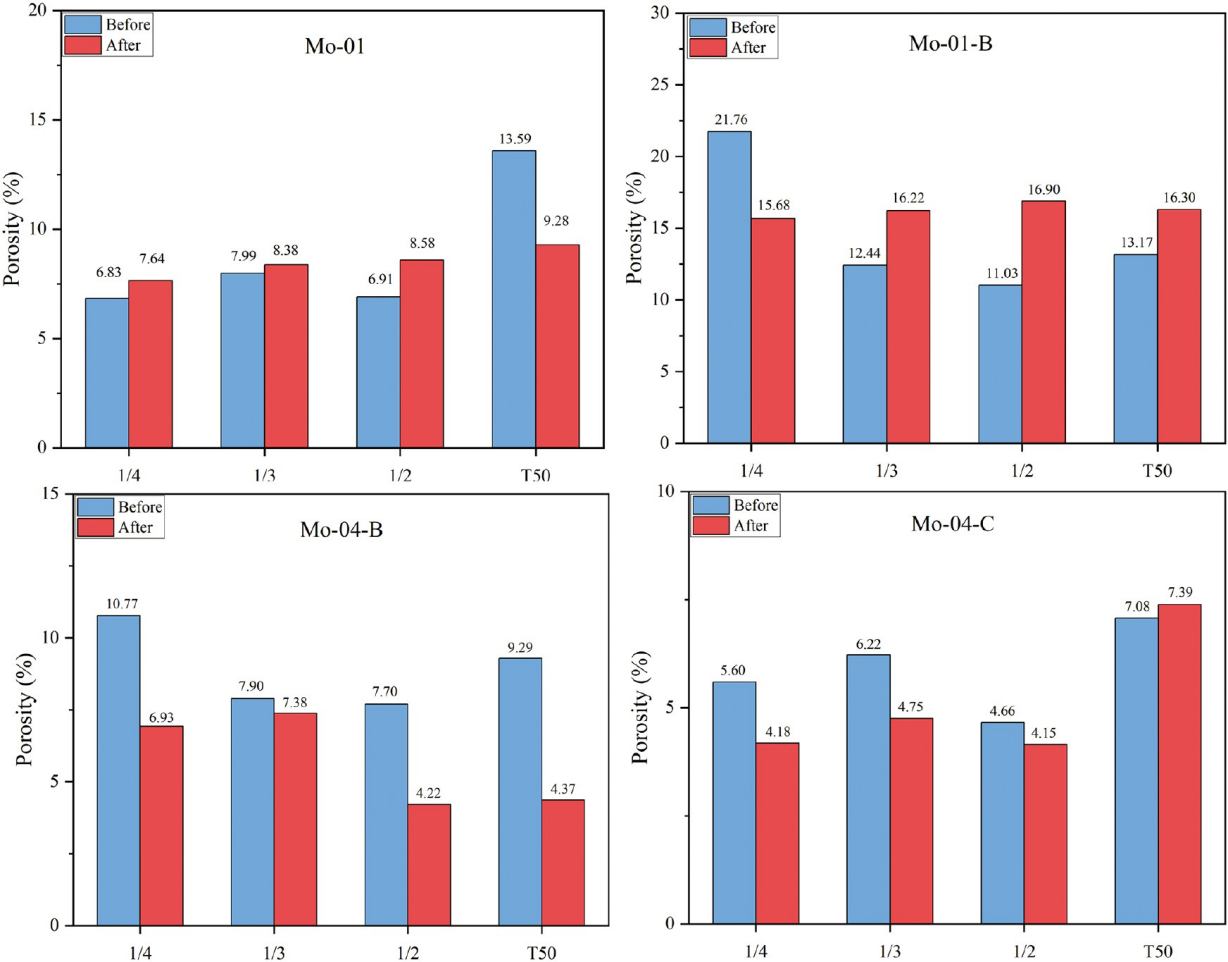
Porosities before and after acid dissolution.

The initial porosities were similar among all samples, except for Mo-01-B, which showed the highest initial value and the greatest increase after dissolution. This sample also displayed the highest surface roughness after dissolution. It contains a significant amount of allochemical grains, such as bioclasts and peloids, whereas the other samples are more homogeneous, dominated by a dolomitic matrix. This feature indicates that samples dominated by carbonate matrix exhibit lower porosity than those dominated by dolomitized grains. This behavior agrees with previously observed trends in Paleozoic carbonate rocks, where grain-dominated fabrics generally retain higher porosity than matrix-dominated ones, reflecting textural differences in these rocks.^37^ Previous studies have shown that porosity and permeability distribution are parameters that directly influence the diffusion coefficient of acid–rock reactions and corrosion patterns.^36,38^

Figure 10 presents the mass loss graph. On the left side of the graph, the variation of mass over time is shown, expressed as the logarithm of mass versus dissolution time (minutes), where a progressive decrease in mass can be observed. A trend line was plotted, and the Table 5 displays the *R* ^2^ values and the corresponding linear equations. The absolute value of the slope was used to represent the acid–rock reaction dissolution rate, following.^34^

**Figure 10:**
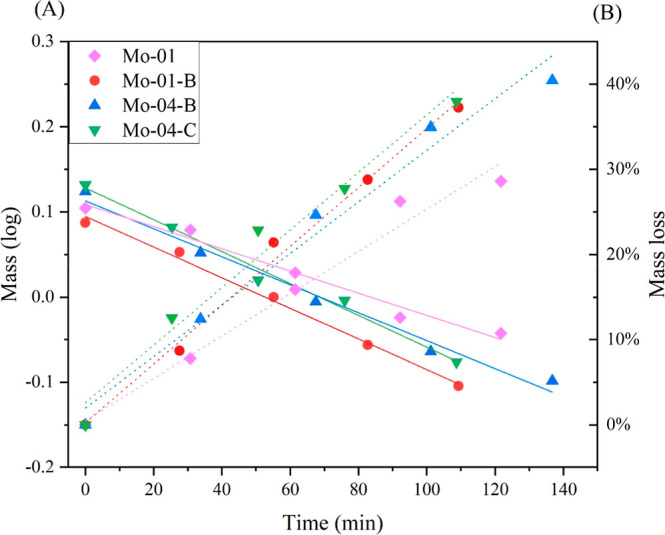
Mass loss during dissolution stages: (A) mass loss represented by the logarithm of mass versus dissolution time (minutes); (B) percentage mass loss versus dissolution periods.

**Table 5:** Linear Regression Parameters for Mass Loss During Dissolution

sample	mass (log) equation	*R* ^2^
Mo-01	*y* = −0.0013*x* + 0.10873	0.973
Mo-01-B	*y* = −0.0018*x* + 0.09465	0.994
Mo-04-B	*y* = −0.00164*x* + 0.11295	0.979
Mo-04-C	*y* = −0.00187*x* + 0.12822	0.984

The samples follow the following order of dissolution rate: Mo-04-C > Mo-01-B > Mo-04-B > Mo-01. The first two samples exhibited very similar dissolution rates, which can be attributed to their comparable chemical compositions, with similar calcium (32.9%, 28.4%), magnesium (17.3%, 20.1%), and silicon (2.9%, 2.5%) contents. XRD analysis indicated mineralogical similarity among them, although sample Mo-01-B shows a peak of magnesian calcite, which is absent in the others, while Mo-04-C exhibits the most intense calcite peak of all. However, it is important to note that the results for Mo-04-C were strongly influenced by the larger crystals that detached during dissolution, leaving relatively large cavities in the mini-plugs and thus affecting the mass loss of these samples.

The samples that exhibited lower dissolution rates (Mo-01 and Mo-04-B) show chemical and mineralogical similarities, with comparable calcium (26.1% and 22.4%), magnesium (18.4% and 15.7%), and silicon (11.1% and 15.3%) contents. The higher silicon content, likely associated with quartz presence, suggested that sample Mo-04-B would present a lower dissolution rate than Mo-01. This expectation is consistent with the XRD results, which revealed calcite peaks in Mo-01, while Mo-04-B displayed only dolomite and quartz peaks. Considering that quartz is highly resistant to dissolution and that calcite is one of the carbonates with the highest dissolution rates in HCl,^9,39,40^ it would be expected that Mo-04-B exhibits lower reactivity toward the acid.

However, petrographic analyses revealed that sample Mo-01 contains quartz crystals with larger grain sizes than those observed in Mo-04-B, which likely increased its resistance to acid attack and reduced the dissolution rate. Furthermore, SEM images (Figure 10) showed that Mo-01 has well-developed dolomite crystals with tabular and rhombohedral habits, exhibiting few structural defects. According to Xu et al.,^34^ larger and less defective crystal aggregates tend to show lower reactivity because smaller crystals have a higher number of grain boundaries per unit volume, and crystal defects promote the formation of etch pits on the surface during the dissolution process. This observation is consistent with the results obtained in this study.

Regarding the microfacies classification, a divergence was observed, each microfacies includes one sample among the highest dissolution rates and one among the lowest. Although the highest and lowest rates correspond to distinct microfacies, this contrast is likely better explained by differences in chemical composition and mineralogical characteristics rather than by microfacies alone.

Figure 10 on the right side, shows the percentage variation in mass loss as a function of dissolution time is presented, divided into four periods corresponding to multiples of *T*50, according to the data in Table 1. The highest mass losses were observed in samples Mo-04-B, while Mo-01-B and Mo-04-C exhibited similar values, and Mo-01 showed the lowest mass loss. For Mo-04-B, dissolution time had a more significant influence on the percentage loss than the dissolution rate, since its rate is only slightly lower than the others (except for Mo-01), but its reaction time was at least 15 min longer, as indicated in Table 1. In contrast, Mo-01 exhibited the lowest mass loss despite having a relatively long dissolution time, indicating a stronger influence of the dissolution rate on the final result.

Figure 11, presents the plots of dissolved Ca^2+^ and Mg^2+^ ion concentrations in the solution resulting from the reaction as a function of dissolution time. The observed trends are similar to those of percentage mass loss, confirming that the mass losses were associated with the acid–rock reaction. The concentrations of calcium and magnesium ions gradually increase with reaction time, indicating that the mini-plugs were continuously dissolved as the reaction progressed, which is also evident in Figure 11.

**Figure 11:**
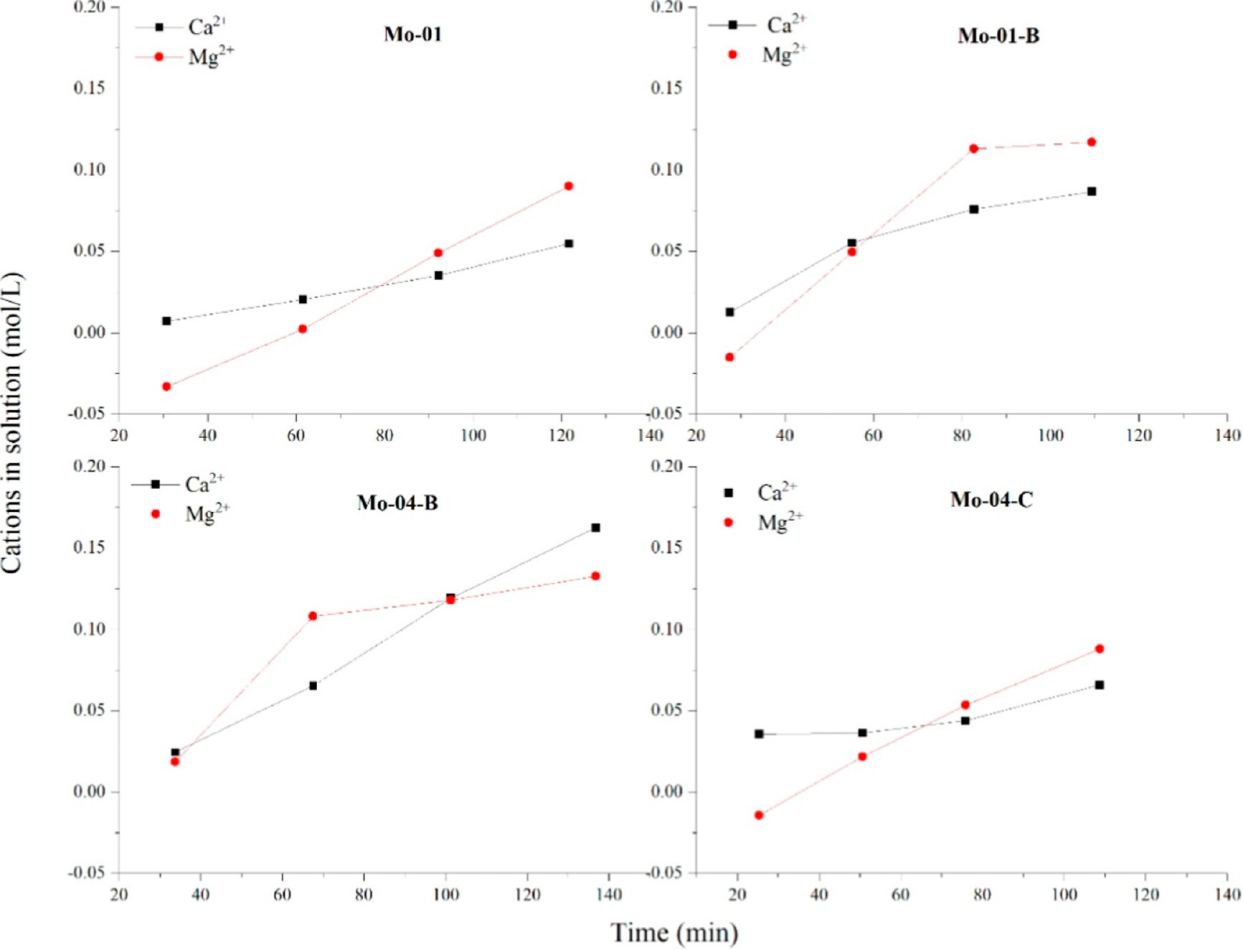
Calcium and magnesium ion concentrations versus dissolution time.

Regarding magnesium, only sample Mo-04-B showed Mg concentrations above the detection limit of the equipment during the first time period. This sample did not exhibit a calcite peak in the XRD analysis, showing only dolomite. For the other samples containing both calcite and dolomite, it is likely that calcite reacted preferentially with the acid, since calcite is more reactive. This is because the Mg^2+^ ion has an ionic radius about 40% smaller than that of Ca^2+^, resulting in an Mg–O bond with greater covalent character and a smaller dipole moment compared to the Ca–O bond.^40–42^

Furthermore, with the exception of sample Mo-04-B, which did not show a calcite peak in the XRD pattern, all other samples exhibited a consistent and noteworthy trend. During the first two sampling intervals, Ca^2+^ concentrations were higher than Mg^2+^ concentrations. However, from the third time point onward, Mg^2+^ concentrations exceeded those of Ca^2+^. Overall, these samples showed a higher rate of increase in magnesium concentration relative to calcium. This inversion from Ca^2+^-dominant concentrations at early dissolution stages to Mg^2+^-dominant concentrations at later stages likely reflects differences in dissolution kinetics between calcite and dolomite. The initially higher Ca^2+^ concentrations are consistent with the preferential and faster dissolution of calcite, whereas the subsequent predominance and steeper increase of Mg^2+^ suggest a progressively greater contribution from dolomite dissolution as the reaction advances.

In contrast, sample Mo-04-B, composed exclusively of dolomite, exhibited a greater overall rate of increase in Ca^2+^ concentration. Although Mg^2+^ showed a higher initial growth rate during the first dissolution interval, its increase appeared to stabilize at a lower rate in the subsequent intervals, which may indicate temporal variations in dolomite dissolution behavior.

When comparing microfacies pairs, the Dpq samples displayed relatively similar trends, suggesting a more consistent mineralogical or textural control. In contrast, the Dc samples showed more distinct behaviors, possibly reflecting greater internal heterogeneity, which may be associated with the presence of calcite grains and calcite cement in Mo-04-C, in contrast to Mo-04-B, where only dolomite grains and minor quartz are observed. Notably, despite belonging to different microfacies, samples Mo-01 and Mo-04-C exhibited the most visually similar dissolution patterns, indicating that mineralogical composition and reactive surface characteristics may exert a stronger influence on Ca^2+^ and Mg^2+^ release than microfacies classification alone.

When comparing the diffractograms of the samples before dissolution with those of mini-plugs 5 and 8 after dissolution, corresponding to the 1/4 and *T*_50_ dissolution periods, respectively (see Supporting Information Figures S3–S6), it can be observed that in the samples that exhibited a calcite peak before dissolution, except for sample Mo-01-B, there was a reduction in peak intensity or even its disappearance, indicating the dissolution of this mineral by the acid.

The full width at half-maximum (fwhm) values of the main diffraction peaks of dolomite, calcite, and quartz for the five samples at *T* = 0, *T* = 1/4, and *T* = *T*_50_ are provided in Table S1 (Supporting Information). An overall increase in fwhm values was observed after dissolution. Peak broadening may be associated with several factors, including reduction in crystallite size and/or the development of microstrain and structural defects, such as dislocations, chemical impurities, or vacancies.^43,44^ Although acidic treatment likely promotes mineral surface degradation, the present data do not allow differentiation between these contributing factors. Therefore, the observed broadening is interpreted as indicative of structural modification.

Overall, the dissolution patterns observed in this study are consistent with previous experimental investigations on acid–carbonate interactions, particularly regarding the preferential dissolution of calcite relative to dolomite and the influence of mineralogical heterogeneity on corrosion morphology. However, the present results further demonstrate that even among samples with broadly similar bulk mineralogical compositions, subtle variations in crystal size, structural defects, and mineral distribution significantly influence dissolution kinetics and ion release behavior. These findings suggest that predictive models of acid stimulation in heterogeneous carbonate reservoirs should incorporate crystal-scale and textural parameters in addition to bulk mineralogical composition. Therefore, the trends identified here may be extended to other dolomitized carbonate systems where small-scale heterogeneities exert strong control on reactive behavior.

## 5 Conclusion

This study evaluated the influence of mineralogical heterogeneity on acid–rock interaction in five dolomitized carbonate samples from the Piauí Formation, Parnaíba Basin. Petrographic, mineralogical, chemical, and porosity analyses were combined with static dissolution experiments and monitoring of Ca^2+^ and Mg^2+^ release.

The results demonstrate that mineralogical composition, crystal texture, and initial porosity strongly control dissolution behavior. Samples with larger crystals developed deeper cavities due to crystal detachment, while porosity changes reflected both dissolution intensity and measurement limitations related to large surface voids. Most samples showed an early dominance of Ca^2+^ release followed by increasing Mg^2+^ concentrations, indicating preferential calcite dissolution and a progressive contribution from dolomite. The exclusively dolomitic sample exhibited distinct kinetic behavior. Postdissolution XRD data revealed peak broadening, suggesting structural modification of the minerals.

Overall, acid reactivity was governed more by mineralogical composition and crystal-scale features than by microfacies classification alone. These findings demonstrate a direct relationship between rock fabric features and dissolution kinetics, reinforcing the need for integrated mineralogical and textural characterization to improve prediction and optimization of acid stimulation strategies in heterogeneous carbonate reservoirs.

The preferential dissolution of calcite relative to dolomite observed here agrees with previous experimental studies on acid–carbonate interactions. However, this study further demonstrates that crystal-scale features, including grain size and structural modification, significantly influence dissolution kinetics even among samples with similar bulk compositions. These findings suggest that small-scale mineralogical and textural heterogeneities should be incorporated into predictive models and may be extended to other heterogeneous dolomitized carbonate systems, particularly in reservoir settings where subtle fabric variations affect stimulation efficiency.

## Supplementary Material



## Notes

Additional details are provided in the Supporting Information, including photographs of the mini-plugs used in the static dissolution tests and their duplicates (Figure S1); images of the mini-plugs before and after static dissolution with HCl (Figure S2); comparative XRD diffractograms of the studied samples before and after dissolution (Figures S3–S6); and full width at half-maximum (fwhm) data of the main diffraction peaks of dolomite, calcite, and quartz before and after acid dissolution (Table S1) (PDF)
