# Supplementary material for: Influence of Mineralogical Heterogeneity on Acid–Rock Interaction in Dolomitized Carbonates from the Parnaíba Basin, Northeastern Brazil: Implications for Reservoir Matrix Acid Stimulation
Source: ACS Omega. 2026 Jun 3;11(23):33807–20. doi: 10.1021/acsomega.6c00330 (PMC13280863; doi:10.1021/acsomega.6c00330)
Supplement: Supplementary file 1 [file ao6c00330_si_001.pdf]

Influence of mineralogical heterogeneity on acid–rock interaction in dolomitized carbonates from the Parnaíba Basin, northeastern Brazil: implications for reservoir matrix acid stimulation

Jéssica Nascimento Pereira,[a,b,], Natalino da Silva Souza[b,c], Igor Alexandre Rocha Barreto[a,b], Renato Sol Paiva de Medeiros[c], José Leão de Luna[c], Pedro Tupã Pandava Aum[c,d] and Cláudio Regis dos Santos Lucas[a,b,c]*

[a]Postgraduate Program in Geology and Geochemistry, Geoscience Institute, Federal University of Pará (UFPA), Augusto Corrêa Street s/n, Belém, 66075-110, Pará, Brazil; [b]Laboratory of Integrated Research in Geoenergy, Federal University of Pará (UFPA), Salinópolis, Pará, Brazil [c]School of Engineering, Federal University of Pará (UFPA), Salinópolis, Pará, Brazil; [d]Petroleum Science and Engineering Laboratory, Federal University of Pará (UFPA), Salinópolis, Pará, Brazil

* Corresponding author

E-mail address: jessica.pereira@salinopolis.ufpa.br

Supplementary material

- **Figure S1**. Mini-plugs during static dissolution tests and their duplicates
- **Figure S2**. Mini-plugs before and after static dissolution with HCl.
- **Figure S3.** Comparison of XRD diffractograms of sample Mo-01 before and after dissolution.

- **Figure S4.** Comparison of XRD diffractograms of sample Mo-01-B before and after dissolution
- **Figure S5.** Comparison of XRD diffractograms of sample Mo-04-B before and after dissolution.
- **Figure S6.** Comparison of XRD diffractograms of sample Mo-04-B before and after dissolution.
- **Table S1.** Full width at half maximum (FWHM) data of the main peaks of dolomite, calcite, and quartz before and after acid dissolution.

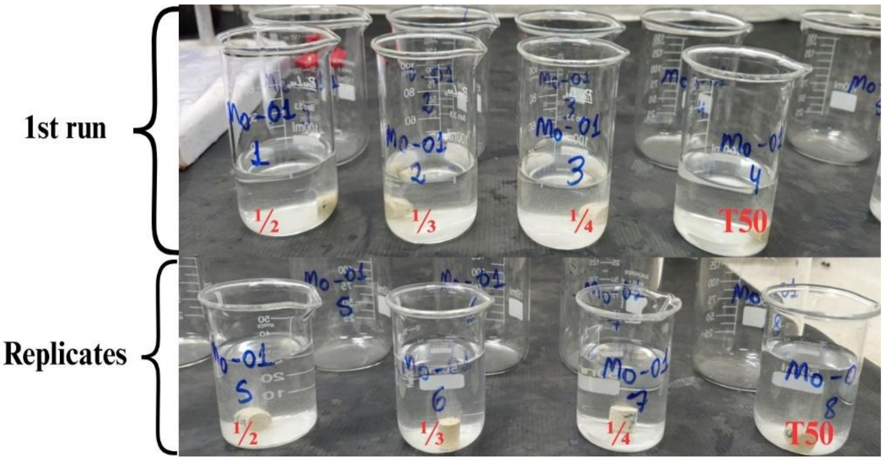

Figure S1. Mini-plugs during static dissolution tests and their duplicates

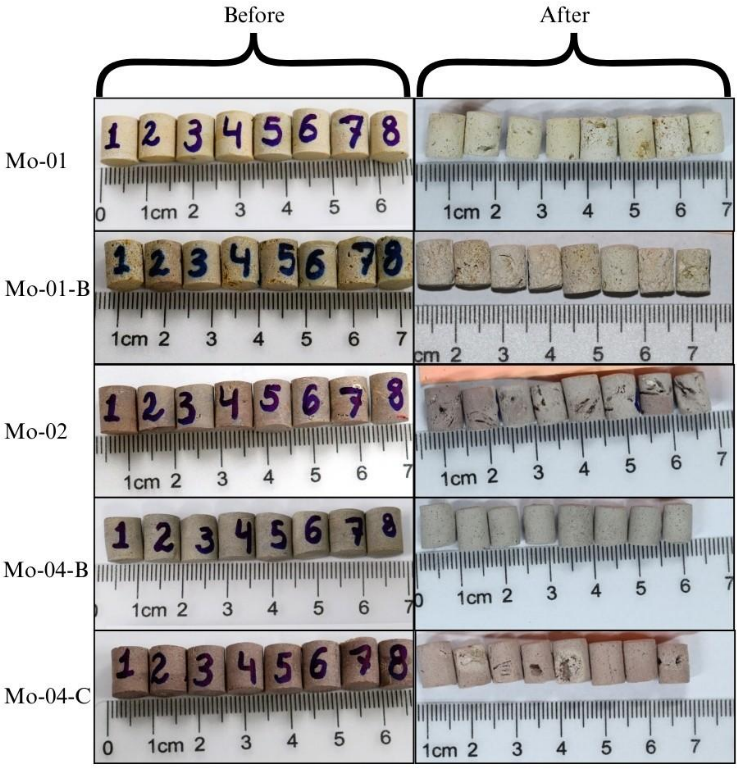

Figure S2. Mini-plugs before and after static dissolution with HCl.

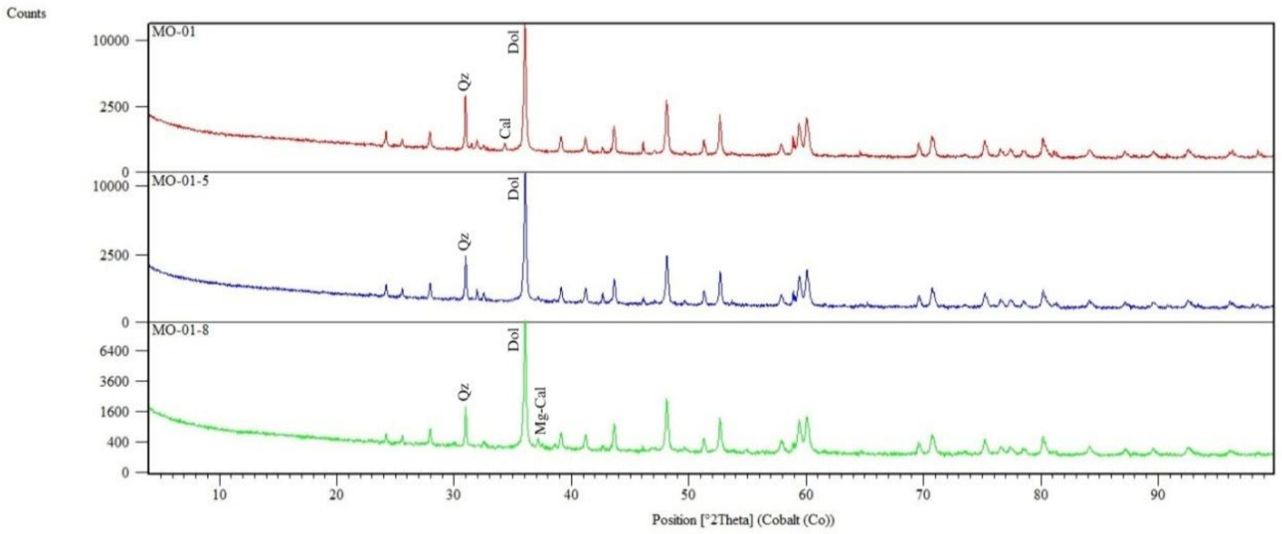

Figure S3. Comparison of XRD diffractograms of sample Mo-01 before and after dissolution.

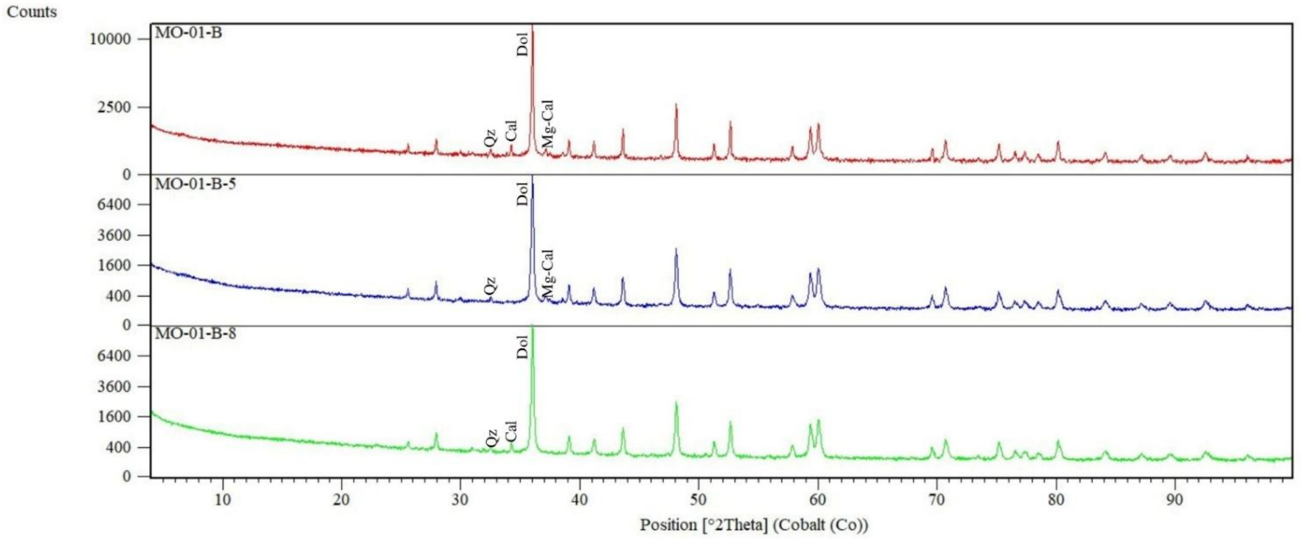

Figure S4. Comparison of XRD diffractograms of sample Mo-01-B before and after dissolution

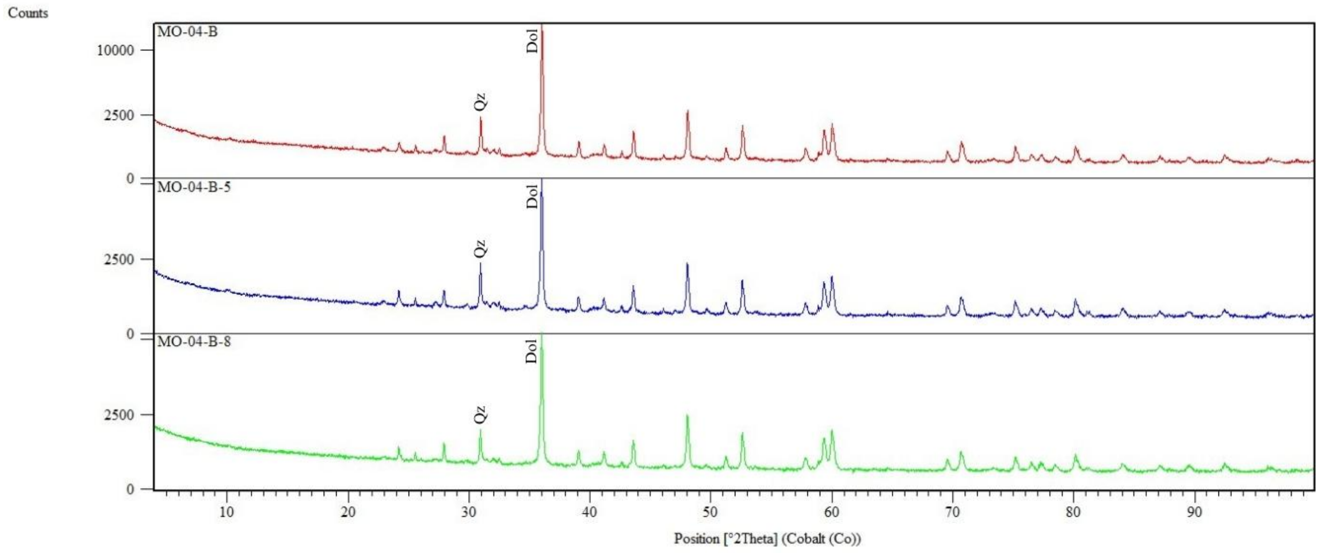

Figure S5. Comparison of XRD diffractograms of sample Mo-04-B before and after dissolution.

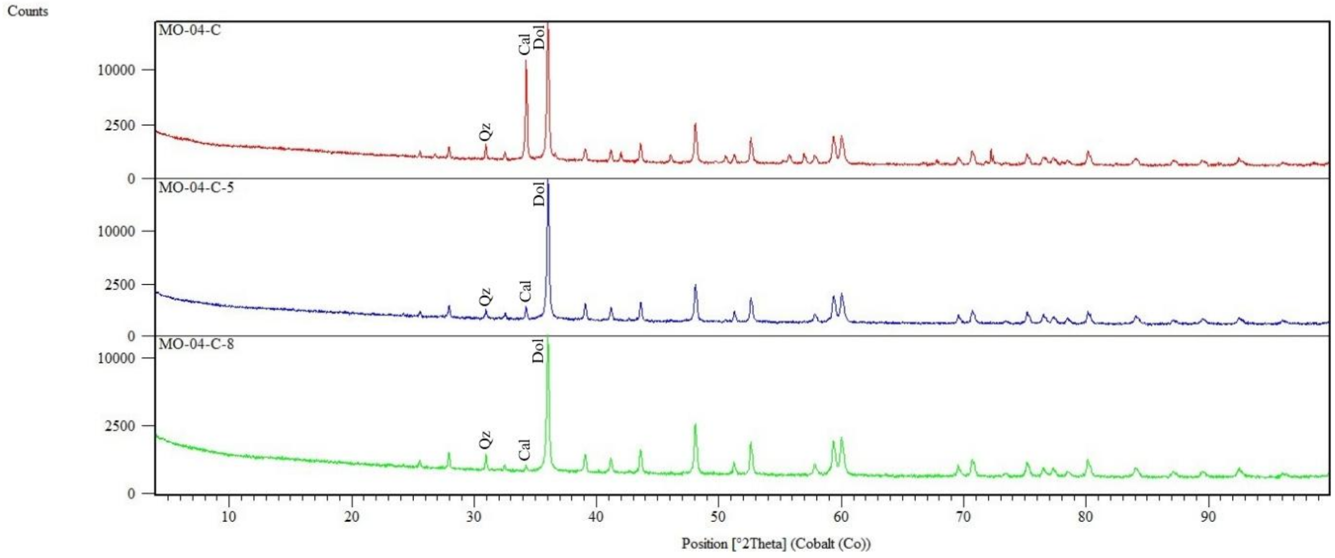

Figure S6. Comparison of XRD diffractograms of sample Mo-04-B before and after dissolution.

Table S1. Full width at half maximum (FWHM) data of the main peaks of dolomite, calcite, and quartz before and after acid dissolution.

Sample	Mineral	FWHM (T=0)	FWHM (T=1/4)	FWHM (T=T_{50})
Mo-01	dolomite	0.1248	0.138	0.153504
	calcite	0.1872	-	-
	quartz	0.0936	0.101	0.102336
Mo-01-B	dolomite	0.1248	0.153504	0.153504
	calcite	0.1248	-	0.102336
	quartz	0.1248	0.153504	0.153504
Mo-04-B	dolomite	0.1248	0.153504	0.153504
	calcite	-	-	-
	quartz	0.0936	0.102336	0.0936
Mo-04-C	dolomite	0.1248	0.1248	0.1248
	calcite	0.0936	0.0936	0.1872
	quartz	0.0936	0.0936	0.0936
